# Review on Nanocrystalline Cellulose in Bone Tissue Engineering Applications

**DOI:** 10.3390/polym12122818

**Published:** 2020-11-27

**Authors:** Nur Ilyana Sahira Murizan, Nur Syahirah Mustafa, Nor Hasrul Akhmal Ngadiman, Noordin Mohd Yusof, Ani Idris

**Affiliations:** 1 School of Mechanical Engineering, Faculty of Engineering, Universiti Teknologi Malaysia, Johor Bahru, Johor 81310, Malaysia; nisahira2@graduate.utm.my (N.I.S.M.); nsyahirah83@graduate.utm.my (N.S.M.); noordin@utm.my (N.M.Y.); 2 c/o Institute of Bioproduct Development, School of Chemical Engineering, Faculty of Engineering, Universiti Teknologi Malaysia, Johor Bahru, Johor 81310, Malaysia; aniidris@utm.my

**Keywords:** nanocellulose, bone tissue engineering, scaffold, acid hydrolysis, mechanical properties, biocompatibility

## Abstract

Nanocrystalline cellulose is an abundant and inexhaustible organic material on Earth. It can be derived from many lignocellulosic plants and also from agricultural residues. They endowed exceptional physicochemical properties, which have promoted their intensive exploration in biomedical application, especially for tissue engineering scaffolds. Nanocrystalline cellulose has been acknowledged due to its low toxicity and low ecotoxicological risks towards living cells. To explore this field, this review provides an overview of nanocrystalline cellulose in designing materials of bone scaffolds. An introduction to nanocrystalline cellulose and its isolation method of acid hydrolysis are discussed following by the application of nanocrystalline cellulose in bone tissue engineering scaffolds. This review also provides comprehensive knowledge and highlights the contribution of nanocrystalline cellulose in terms of mechanical properties, biocompatibility and biodegradability of bone tissue engineering scaffolds. Lastly, the challenges for future scaffold development using nanocrystalline cellulose are also included.

## 1. Introduction

Bone damage can be caused due to aging, tumor, injuries, fracture and accidents [1]. In the human body, bone has a special capability in self-healing, where it regenerates the tissue naturally with time. However, the self-healing properties are only limited to the smaller capacity of bone. Hence, the healing process for large bone damage may take time and difficult for the tissue to regenerate [2]. Hence, clinical treatment is needed to help the healing process. For the past decades, bone cement [3] and bone grafting [4,5,6] have been widely used in treating bone damage. Unfortunately, these two techniques appeared with some drawbacks such as short lifespan, limited donors and risk of bacterial and virus transmission that has led to rising in effort among researchers to find a new way of tackling bone damage [7]. The new technology that has come of interest is called tissue engineering.

It has been reported that modern tissue engineering has started in the late 1970s, but the term “tissue engineering” only be coined in 1987 [8]. In definition, tissue engineering is a modern scientific discipline of clinical treatment that applies engineering and life science principles together. In some studies, tissue engineering is also known as regenerative medicine [9]. This method uses living cells, biocompatible materials, with suitable biochemical and physical factors, as well as a combination thereof, to create tissue-like structures [10]. The aims are to restore, regenerate and heal the damaged tissue with the presence of scaffolds [11].

In further, a scaffold is a framework that use to attach and to proliferate the cells [12] and also to form the extracellular matrix (ECM) for the regenerative process [13]. Scaffold also works as cell carriers, drug delivery such as growth factors and antibiotic and carrier for bimolecular signals [14]. In order to guarantee the successful function of a scaffold, it must possess the following requirements that include:

1. biocompatibility and biodegradability properties [15,16];
2. mechanical rigidity and flexibility [17];
3. suitable surface topography and chemistry [7];
4. high surface area to volume ratio [18,19,20,21];
5. suitable pore size and large porosity [22,23].

To not forget, the correct selection of biomaterials in developing scaffolds has a big impact on the biocompatibility and mechanical properties of the scaffold [24]. From past studies, the most common used material is polymer-based biomaterials such as PLA [23], PCL [25], PPF [26], PVA [27,28] and PEG [7]. Despite many biomaterials, tissue engineering is still facing some limitations were to translate the theoretical concept into reality seems to be unrealistic with the inability of engineered materials to mimic the natural properties of tissues [29]. Therefore, researchers have come out with the idea to includes nanoparticles as reinforcing agents [30] and nanofillers [31] in the scaffold in order to enhance mechanical and biocompatible performances.

Nanoparticles are characterized by nanoscale dimension, enabling them to develop critical physical and chemical characteristics that enhanced their performance and therefore make them beneficial for a wide range of applications. Recently, nanoparticles have widely been used in tissue engineering [11]. Nanoparticles can be divided into organic and inorganic nanoparticles. Organic materials come from polymer and natural sources, while inorganic materials consist of metallic nanoparticles such as gold, silver and magnetic nanoparticles consist of metal oxides. The problem with inorganic nanoparticles is that it is not biocompatible for the human body even though it offers better mechanical strength [11]. Hence, to solve this problem, organic nanoparticles derived from plants have been used in tissue engineering. Recently, plant-based cellulose nanoparticles have been discovered as the most abundant type of renewable organic source on Earth. In tissue engineering, nanocellulose act as cell adhesion for cellular attachment [15,16]. It has been reported that nanocellulose provides biocompatible and mechanical properties, but their biodegradable ability still under discussion.

Consequently, many reviews on the application of cellulose in biomedical applications have been reported. However, most reviews were focusing only on general knowledge of nanocellulose or only on bacterial cellulose in tissue engineering applications [13,32,33,34]. There are some reviews that have focused on nanocrystalline cellulose (NCC) and its application in various fields [20,35]. However, to the best of our knowledge, there is still no review that is concentrating on the application of NCCs derived from agricultural waste for tissue engineering applications.

To close the gap, this review provides an overview of the recent development of NCCs as nanomaterials in bone tissue engineering scaffold applications, focusing on attributions of NCCs in the development of bone scaffolds. In detail, a concise introduction of tissue engineering principles and its requirement is provided to highlight the potential of NCCs in this field. The literature on nanocellulose, which is nanocrystalline cellulose (NCC) as if their sources, extraction methods is reviewed to give more understanding about nanocellulose. The crucial emphasis is on the contribution of NCCs in terms of mechanical property; its biocompatibility and biodegradability properties are highlighted with the challenge for the future development of bone scaffolds.

## 2. Bone Structure and Its Properties

**Figure 1:**
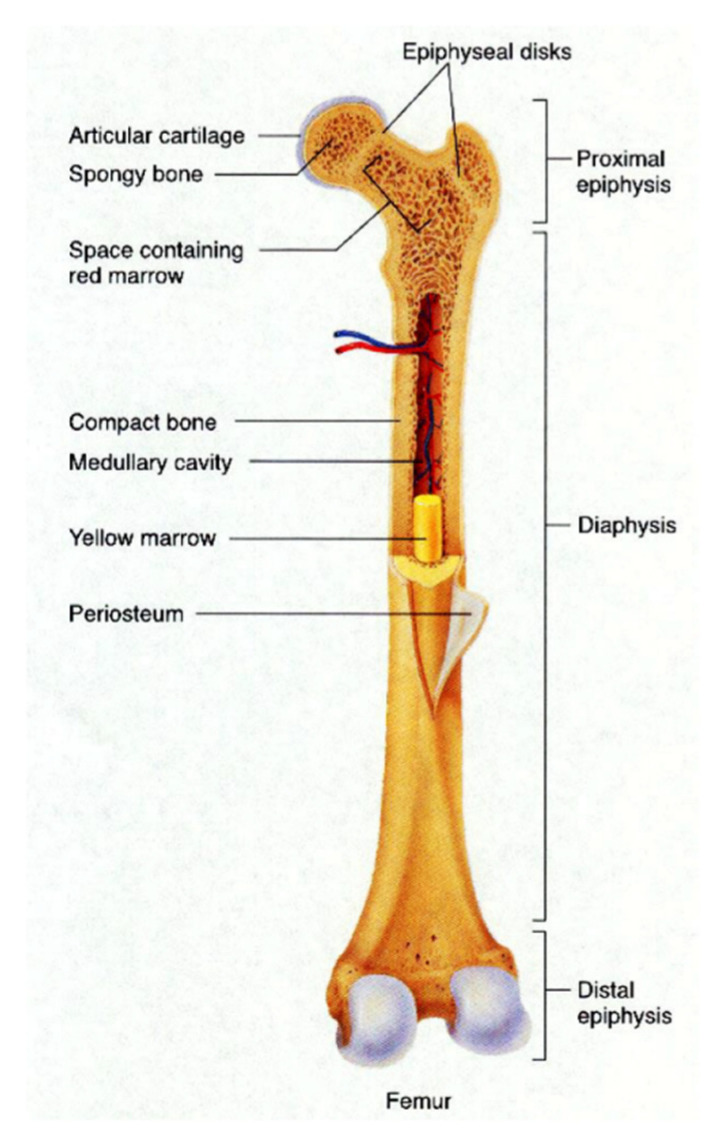
Femur bone structure [36].

Bone is part of the skeletal system that provides support and movement of the body, as well as protecting the organ inside the body. Bone structure is categorized into two main structures; compact bone and spongy bone, as shown in Figure 1. Compact bone or cortical bone is a hard-outer layer of long bone found in the diaphysis part. Spongy bone is a thin shell that surrounding the trabecular compartment in the epiphyses and is also known as trabecular bone because it is composed of trabecula networks. Most of the long bone has bone marrow that produces and supply red blood cells and white blood cells for the human body. The yellow bone marrow is located within a compact bone cavity that represents fatty tissue, whereas the red bone marrow represents hematopoietic tissue is located in the spaces between trabeculae of spongy bone [36]. Both tissues contain a high number of vascularized networks in order to supply enough nutrients and to remove waste [37].

Compact bone is a dense, strong and durable structure that provides protection to the inner layer of bone with high compressive strength ranging from 100 MPa to 190 MPa, but it contains low porosity [38]. It also makes up to ~80% of the total bone mass in adults [39,40]. The spongy bone that accounts for another 20% of total bone mass has high porosity ranging from 40% to 90% to allow better penetration of vasculature. However, it is low in mechanical strength compared to the compact bone, with only 7–10 MPa of compressive strength [38]. The properties of human bone depend on the part of the body and age. This is because each part of bone posses’ different mechanical properties. As we age, the bone becomes more brittle, and mass and density are reduced due to loss of calcium and other minerals [41].

## 3. Cellulose

**Figure 2:**
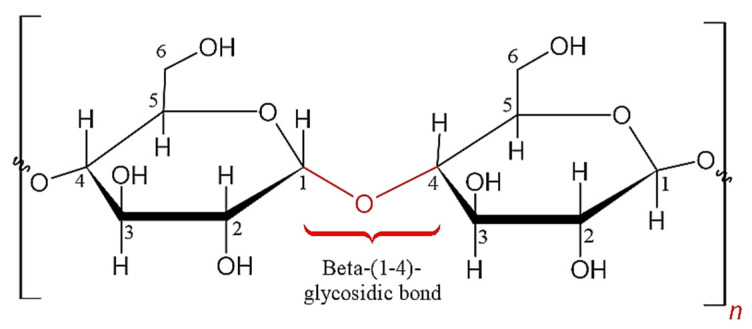
Single cellulose chain repeat unit depicting two glucose units with a Beta-(1-4)-glycosidic linkage.

The increased demands for products made of renewable and sustainable resources have led to the discovery of cellulose. Cellulose is an organic compound with a polysaccharide formula consisting of a linear chain of several hundred to over ten thousand D-glucose units that are linked by Beta-(1-4) of glycosidic bonds as shown in Figure 2 [35,42,43,44]. For each unit of D-glucose, it is featured with six hydroxyl groups and two glycosidic bonds. The extensive hydroxyl function has restricted the polymer chain flexibility and provides greater rigidity to the polymer [38]. It is an important structural component in primary plant cell walls.

Cellulose can be derived from wooden plants, non-wooden plants and some kind of algae and bacteria. Each type of plant undergoes different mechanical or chemical treatment in order to isolate cellulose. For the past few decades, cellulose-based biomaterials have been discovered and achieved remarkable applications in various fields such as paper and paperboard, composite, food, hygiene and absorbent products, emulsion and dispersion, medical, cosmetic and pharmaceutical. Recently, it has come to an interest among researchers to study the applications of cellulose in tissue engineering. This is because of the encouragement in using green materials, in which cellulose is abundant high-performance materials that are almost inexhaustible sources on Earth [45]. It is also easy to be processed into many arrays of materials.

Furthermore, cellulose can be derived into micro to nano-sized cellulose particles. Different types of sources and derivation methods will result in different cellulose particles, which are bacterial cellulose (BC), fibrillated cellulose (FC) and crystalline cellulose (CC), as shown in Table 1. These cellulose particles have been studied extensively on their potential in tissue engineering application based on their tunable chemical and physical properties, biocompatibility and biodegradability properties [46,47].

### 3.1. Bacterial Cellulose

Bacterial cellulose (BC) is sort as pure cellulose with a highly porous structure and exhibits extensive water retention [15]. Bacterial nanocellulose (BNC) is produced by building up of low molecular weight of sugars and alcohols by bacteria (named *Gluconacetobacter xylinus*) for a few days up to two weeks. This process is called a bottom–up process [20]. In terms of morphology, BNC is reported to be consisting of more than one (1) micrometer length and at a range of 30–50 nm width with 65–79% of crystallinity index [13,33]. In clinical treatment, BC offers a unique combination of mechanical properties, interconnected porosity, biocompatibility, and the ability to absorb and hold large quantities of water [33]. BNC is said to offer better mechanical properties compared to FC and CC and has been widely used as biomaterials for biomedical applications.

### 3.2. Fibrillated Cellulose

The second type of cellulose particle is fibrillated cellulose (FC). FC can be derived from wood pulp into two forms, microfibrillated (MFC) cellulose and nanofibrillated cellulose (NFC). Commonly, the wood pulp is delaminated by mechanical pressure and has been treated chemically or enzymatically to produce FCs in the form of nano- or micro- sizes. It has been reported that the size of FC is in the range of 4–100 nm wide and 0.5–50 µm-long [49]. FCs can also be derived from potato peel, sugar beet and hemp. FC is usually used in nanofibrous structures with other biomaterials in scaffold design.

### 3.3. Crystalline Cellulose

The other form of cellulose particles is in the form of crystalline cellulose (CC), which is also available in two sizes, micro- and nano -sizes. Focusing on nanocrystalline cellulose (NCC), it is also referred to as cellulose nanocrystal (CNC) [46,53], nanowhisker cellulose (NWC) [33,54] and rod-like shaped cellulose. The production of NCCs is reported in the late 1940s and early 1950s by Ranby and Ribi [55]. Since then, NCCs derived from many sources has been studies in term of production methods and their properties. The key application of this structure involves their use in reinforcement systems, and it can be used to replace carbon nanotubes as fillers. This is because of their relatively low cost of production, easy obtainment process, low density, non-abrasive nature, biocompatibility and biodegradability.

From the past studies, NCCs have been derived from cotton, wood, non-wood fiber, some form of algae, tunicate and also from bacterial cellulose by treating the sources with concentrated acid solutions. Above all, NCCs can also be found in lignocellulose biomass, which is agricultural residues/wastes that are rice husk, rice straw, wheat straw, empty fruit brunch, corn stalk, and many others. In Malaysia, it has become an interest among researchers to isolate NCCs from agricultural wastes due to massive production of wastes and also as an initiative to reduce open agricultural burning. Apart from this, agricultural waste is an underutilized byproduct produced and significantly abundant.

In term of morphology, NCCs is the smallest size of cellulose particles that comes in a size of 3–5 nm-diameter and 0.05–0.5 µm length [56]. NCCs have become an interesting nanomaterial after BNCs as they offer good reinforcement capability with the ease to be tailored following application needs. Therefore, this review is focusing on crystalline cellulose in bone tissue engineering applications.

### 3.4. Derivation of Nanocrystalline Cellulose

Due to outstanding properties offered by nanocrystalline cellulose (NCC) and it has a high possibility in future applications, the study on its derivation process from lignocellulosic biomass is very interesting, but in this review, procedures will not be discussed in details as many related reviews have discussed on it [20,57,58]. Generally, to obtain NCCs, the non-cellulosic components such as lignin, hemicellulose and other impurities are detached first. Then the NCCs are extracted by using various methods such as acid hydrolysis or enzymatic hydrolysis.

**Figure 3:**
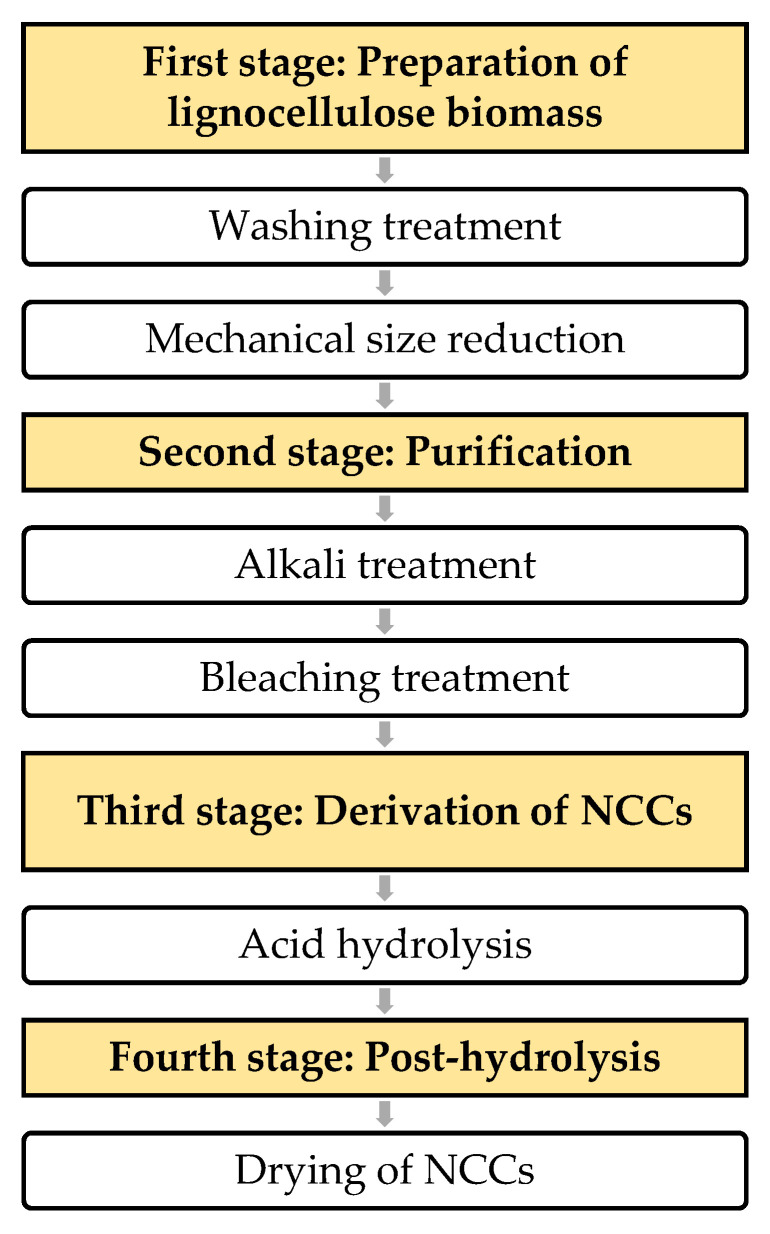
Summary of the isolation process of nanocrystalline cellulose (NCC).

Nevertheless, much discussion will be on the effects of acid hydrolysis conditions on the productions of NCCs. Enzymatic hydrolysis is out of the scope of this review. Notably, acid hydrolysis is an established derivation method and has been used widely compared to other methods. Figure 3 shows the summarization of the derivation process of NCCs that consists of four stages. Each stage is briefly discussed.

**Stage 1: Lignocellulosic biomass preparation and washing treatment**

The first stage of the NCC isolation process involves washing treatment and mechanical grinding. Generally, lignocellulosic biomass (LB) is first dispersed and soaked in a solvent such as tap water, distilled water (DW) or deionized water (DIW) to remove dirt and aqueous soluble substances [57]. Then, the cleaned LBs are air-dried until it is fully dried or can also use the oven-drying method at temperatures ranging from 60 °C to 80 °C for 24 h. After this, the dried-cleaned LBs are subjected to mechanical processes such as cutting, milling and grinding in order to transform dried LBs into powder form. The powder form of NCCs is then sieved using a sieve machine or a shaker with 35 mesh until 80 mesh to obtain fine particulate fibers [19]. The powder form of fibers provides a bigger contact surface area of active groups of cellulose fibers and chemical, which also lead to an increase in the reaction rate of alkali and bleaching treatment in stage 2.

Before proceed to the second stage, washing pretreatment is required to remove wax, pigments, oils and other impurities lying on the external surface of cell walls. This step is also called the dewaxing process. The alternative way for the dewaxing process is using the Soxhlet extraction method with 2:1 of toluene/ethanol [59] or benzene/ethanol [60] mixture at 90 °C for 12 h. Then, the dewaxed fires are oven-dried once again at the same condition as the previous one. The selection of mixture actually depends on the type of cellulosic plants.

**Stage 2: Purification treatment**

There are two main treatment processes involve in stage two (2). Continuing from stage 1, the dewaxed powder of NCCs undergo an alkali treatment or mercerization process to remove hemicellulose and silica compounds [52]. The NCCs powder is subjected to a fairly concentrated base solution to remove alkali-soluble substances such as aqueous potassium hydroxide (KOH) or sodium hydroxide (NaOH) at 4 until 20 wt % with a stirring condition of 80 °C to 90 °C for 1–5 h [59,61]. The aim of this step also is to expose the cellulose by removing the outer layer composed of hemicellulose and silica compound [57].

Next, acid-chlorite treatment, also known as the bleaching process or the delignification process that has been broadly used and has become an important process of isolating the NCCs. This treatment is used to dissolve lignin, and other unwanted impurities left after alkali treatment. The process is conducted by combining three types of solvents, which are distilled water, sodium chlorite and acetic acid, and mix into products from alkali treatments. The solution is mechanically stirred at a temperature range of 60 °C to 80 °C for 4 h until 12 h [62]. The end white solid products are then dried in an oven at 50 °C, which indicates that the lignin and other unwanted impurities have been successfully removed.

However, some studies have conducted bleaching treatment first before alkali treatment [20,59], and some are vice versa [45,63]. There is no study on the effect of conducting different steps of alkali-bleaching treatment or bleaching-alkali treatment.

**Stage 3: Acid hydrolysis**

**Figure 4:**
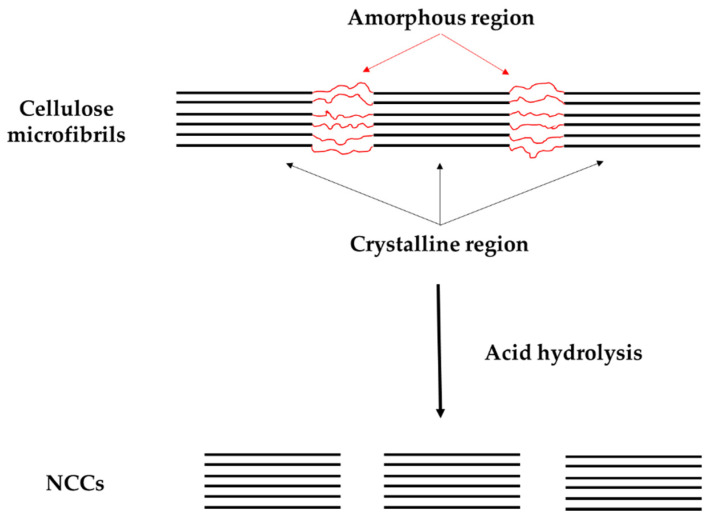
Schematic diagram of acid hydrolysis to extract nanocrystalline cellulose (NCC).

The critical part of deriving the NCCs is on acid hydrolysis process. Theoretically, during the hydrolysis reaction, the amorphous region is broke down by acidic conditions while crystalline regions are mostly insoluble in acids, as shown in Figure 4 [64]. In terms of procedure, the treated NCCs powder is mixed into an aqueous acid solution, which referring to acid to cellulose powder ratio in unit mL per gram. The hydrolysis is conducted under stirring conditions at a certain temperature and period of time. Suggested that the hydrolysis is stopped by diluting the mixture with water (10-fold water) and centrifuge the resulted NCC gel. After this, neutralization is performed using distilled water to remove free acid from the dispersion [19,63]. Some studies also include additional steps, such as filtration, centrifugation or ultracentrifugation, as well as mechanical or ultrasound disintegration [65].

**Figure 5:**
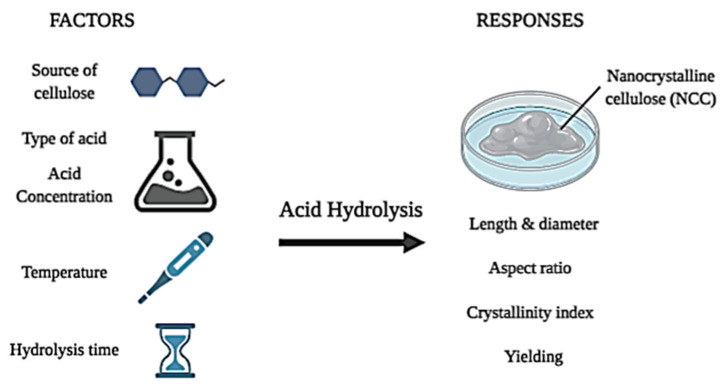
Controlling factors in acid hydrolysis process that affecting few responses (created in Biorender.com).

Continuing from stage 2, the pretreatment could bring a big influence on the efficiency of following acid hydrolysis. In addition, in the acid hydrolysis process, there are few main factors that need to be put into consideration, including the type of lignocellulosic sources, type of acids and their concentration, reaction temperature and hydrolysis reaction time, as shown in Figure 5. All these factors affect the morphology [61,62], crystallinity index [66,67] and yielding [63] of NCCs. All of these effects are obtained through the characterization of NCCs after derivation.

The first controlling factor is the type of acid and its concentration. It has been reported that there are a few acids that can be used for hydrolyzing the NCCs, which includes hydrochloric acid (HCl) [63], nitric acid (HNO_3_) [63], maleic acid, hydrobromic acid (HBr) [51] and the most extensively used acid is sulfuric acid (H_2_SO_4_) [54,59,68] with concentration ranging from 30–65% depending on the type of lignocellulosic sources. The hydrolysis is conducted under temperature that ranges from 30 °C to 100 °C with hydrolysis time varies from 20 min to 4 h. Generally, lower acid concentration and lower temperature during hydrolysis reaction will need longer hydrolysis time for the reaction to be fully completely which also depending on the type of sources and acid. It has been suggested that optimum acid hydrolysis condition for kenaf bast fibers is by using H_2_SO_4_ with 65 wt % concentration at 45 °C and 40 min of reaction time [49].

The wide use of sulfuric acid (H_2_SO_4_) is because as the reaction time increase, esterification or surface sulphation is highly induced in order to introduce a higher amount of residual sulfate groups on the NCC surface, enabling thermal stability [62]. Even so, the hydrolysis reaction period has a bigger influence on the production of NCCs. Hydrolysis time affects the crystallinity and yielding of NCCs proportionally while reciprocally affect the aspect ratio (L/D) [19,57]. Most studies focused only on crystallinity index results than aspect ratio because a bigger value of crystallinity increased the reinforcing effect of NCC, which led to a better mechanical performance of structure [62,63]. However, the aspect ratio of NCCs also plays a vital role in their reinforcing capabilities. It is also as important as the crystallinity of NCCs as it reflects the ability to distribute mechanical stress in the interface matrix or filler. The higher aspect ratio gives greater reinforcement and mechanical properties in the nanocomposite to which they are applied [54].

In addition, the thermal stability of NCCs is also donated by good crystallinity and yielding of NCCs [69]. Prolong hydrolysis time has increased the molecular weight degradation as well as the amount of shortened cellulose chains, which then lowered the thermal degradation energies [57]. Therefore, the longer reaction time only leads to further reduce the NCC yielding due to further digestion of crystalline domains and further hydrolysis of amorphous cellulosic regions.

**Stage 4: Post-hydrolysis**

After completing the acid hydrolysis, post hydrolysis that involves drying process is conducted. There are a few types of drying that can be used, which are oven-drying [70], freeze-drying [59], spray-drying [56] and supercritical drying [57]. However, the most technique used is oven drying because it is simple and easy to conduct. To dry NCCs with an oven, the temperature is set at 60 °C to 110 °C for 24 h. However, the freeze-drying method is also widely been used as it needs low temperature by putting the NCCs into the refrigerator or spray them with liquid nitrogen [59]. Then, the frozen NCCs are transferred into the freeze dryer at a temperature of −80 °C for 24–72 h [71]. This method is also called lyophilization [35].

## 4. Contribution of Nanocrystalline Cellulose in Bone Tissue Engineering

The extraordinary mechanical and physicochemical properties of NCC has caught the attention of many researchers to use it in biomedical applications. NCC has been used a lot in biomedical applications, namely wound dressing, artificial skin, artificial blood vessels, vascular graft, and scaffold for tissue engineering. This review is only reviewing applications of NCCs in tissue engineering, focusing on bone applications, and the other applications are beyond the scope of this review. In bone scaffold tissue engineering, NCCs are used as reinforcing agents, and nanofillers [72,73], additives [61] and some studies used NCCs as biomaterials [68,71,74] with enhanced mechanical and biocompatibility properties of bone scaffold tissue engineering. The enhancement of scaffold properties by inducing NCCs has led to bone regeneration.

In developing the bone scaffold tissue engineering, there are few controlling factors that should be considered, which are:

1. biomaterials with suitable additives and modification, and
2. fabrication process.

These two factors will affect the:

1. mechanical properties:
2. biocompatibility;
3. biodegradability; and
4. morphology of scaffold; this effect will not be discussed in this review.

### 4.1. Mechanical Properties

Scaffold experience different kinds of loads in vivo once being transplanted into the animal or human body. The different loads experienced by scaffold include compression, tension, shear, torsion, bending, and biomechanical/physiological loadings. Therefore, it is important to consider mechanical properties in scaffold development. The scaffold must adequate mechanical strength according to which anatomical site it is being implanted [75]. In addition, it must provide and retain sufficient mechanical support during cell proliferation and during tissue regeneration without causing deformation to new tissue [67]. Natural types of bone consist of two parts; cortical bone, which is the hard bone and trabecular bone or also called cancellous bone, that consists of spongy tissue. Both types of bone own different mechanical strengths. Young’s modulus and the compressive modulus of cortical bone are 15–20 GPa and 100–200 Mpa, whereas the trabecular bone ranges between 0.1 and 2 GPa and 2–20 MPa, respectively [76]. In addition, the mechanical integrity of scaffold can be affected by other factors, also such as pore size, pore interconnectivity, porosity, biomaterials composite, and material density [27].

To tailor the mechanical properties of scaffold, according to a specific application, researchers has included organic nanomaterials such as MCC/NCC as reinforcement agent and biopolymer filler replacing carbon tube. The inclusion of MCC/NCC is also expected to enhance the mechanical properties of the scaffold. This concurred with Lee et al. [51], who developed a nanocomposite film using microcrystalline cellulose (MCC) with polyvinyl alcohol (PVA). It showed that the tensile strength has increased with the increase of MCC loading. The study has been expanded by Cataldi et al. [47], who developed a composite scaffold-based PVA containing various amounts of nanocrystalline cellulose (NCC). From the study, the author has evaluated the tensile properties, which is stress at the break that resulted in 73% increment of stress at the break as 5 wt% of NCC included into the PVA biomaterials. However, the excessive amount of NCC has reduced the tensile stress due to the agglomeration of filler inside the matrix. Another favorable biopolymer is polylactic acid (PLA). Unfortunately, it has been reported that PLA alone is the lack of mechanical integrity, which is lower than the native tissue. Hence, to solve this problem, PLA has been grafted with maleic anhydride coded as MPLA. But the mechanical strength is still below requirement, so NCC was incorporated into MPLA scaffolds [77]. These inclusions of NCC has proved to enhance the tensile strength by 85% at the optimum amount (5 wt %) of NCC. A similar result of mechanical enhancement by the inclusion of NCC into the biomaterial was obtained by Zhang et al. [78], but in this study, NCC was grafted with polyethylene glycol (PEG).

In the in vivo condition, compression frequently occurs in bone scaffold implantation [79,80], while for other applications like for skin or cartilage, the tensile test is more important [75]. Hence, researchers have put an interest in studying the compressive strength of scaffolds as in real application in the human body; scaffolds face compression effect from the host body more than tensile effect [79]. So, Eftekhari et al. [81] have alternatively developed the scaffold with nanocomposite containing poly-L-lactide acid (PLLA), hydroxyapatite (HA) and MCC. The results of the compression test indicated that the incorporation of MCC and HA nanoparticles improves the compressive strength and modulus of the scaffold. This significance may be attributed to the improvement of interfacial bonding between the reinforcement and the polymer by increasing hydrogen bonding between the reinforcing agents and the PLLA matrix. Similar findings were also obtained by Aleman-Dominguez et al. [73], which material designs include MCC-filled PCL reported to obtained compression modulus in the interval of values for spongy bone and Li [82] that use NCC aerogel based scaffold. Other than this, PVA biomaterials have been used to develop scaffolds by Kumar et al. [27] with the incorporation of n-HA and NCCs. This nanocomposite scaffold improves the compression strength from 0.40–2.09 MPa and the compressive modulus from 0.32–16.01 MPa. This study has been continued with additional ovalbumin (OVA) [83]. The effect of NCC in enhancing mechanical properties at optimum content is still there, but the magnitude of compression strength and compression modulus was reported to be below the previous study. The mechanical profile demonstrates irregular behavior in increasing the mechanical properties due to various parameters such as material processing, porosity and pore interconnectivity. Also, the inclusions of OVA and n-HA has affected the hydrophilic behavior that influences the compressive stress–strain behavior has been reduced that range from 0.19–0.37 MPa depending on concentration of NCC and n-HA. Next, Luo et al. [46], has developed as PLA/NCC in situ nanocomposite porous scaffold. From the study, the compression modulus of scaffold has increased by 368% for 0.8 wt % of NCCs-filled PLA compared to that PLA scaffold alone.

From these reviews, it has been proved that NCCs offer the capability to enhance mechanical properties, tensile and compression of bone scaffolds at the optimum amount of NCC-filled base biomaterials. The amount of NCCs included and been tested is ranging from 0.5 wt % to 20 wt %. Yet, the over the amount of MCCs or NCCs has caused the biocomposite of the scaffold to become brittle [81]. There is still no study on optimizing the amount or concentration of NCC to be included in biomaterials for bone tissue engineering scaffold applications. Table 2 shows recent studies on the contribution of NCCs inclusion in bone scaffold material design in terms of mechanical properties.

### 4.2. Biocompatibility Properties

Biomaterials are considered biocompatible when they have the ability to be nontoxic to the living tissues and also can appropriately stimulate the host response in the human biological system. Biocompatibility is one of the requirements in the materials design of bone tissue engineering scaffold for satisfactory performance. The cytotoxicity of biomaterials is determined by the capability of living cells to adhere, proliferate and integrate wall with host tissues. The studies are comprised of in vivo study that involves living biological entities within the organism and an in vitro study, in which the living cells derived from humans or animals is used in the lab.

**Figure 6:**
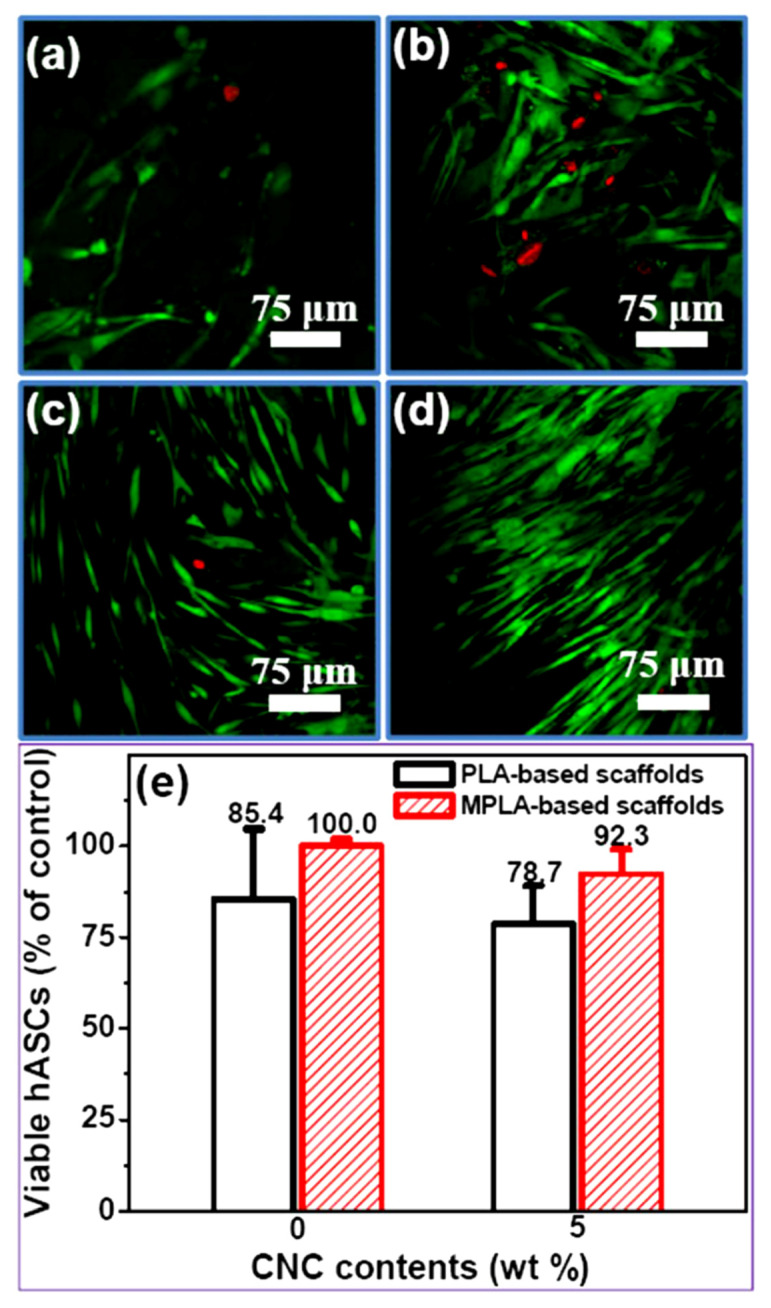
Result of hASCs cultivation on polylactic acid (PLA)/ nanocrystalline cellulose (NCC) and polylactic acid grafted with maleic anhydride (MPLA)/NCC after 7 days. Fluorescence micrograph of (**a**) PLA, (**b**) PLA/NCC-5, (**c**) MPLA, (**d**) MPLA/NCC-5 and (**e**) proliferation viability of cells (reproduced from [77] with permission. Copyright © 2013, American Chemical Society).

Shaheen et al. [53] conducted a cell culture MTT assay using MG63 Osteoblast cells for scaffolds that consist of chitosan and alginate filled with NCCs. At early culture time, the cells have started to grow inside the scaffold pores. After 72 h, cells have seen to attached tightly through their filopodium and lamellipodium inside the pores of a 3D structure. The dense cells grew inside and outside of pores as clusters with highly interconnected 3D network structure. So, this result indicated that the scaffold has shown obvious proliferation tendency and was nontoxic and suitable for attachment and growth of MG63 Osteoblast. On the other hand, the viability of M058K cell seeded on PLA and NCC-filled PLA by Luo et al. [46]. The Alamar blue assay is used, and the activity of cells is recorded on days 3, 6 and 12. It resulted that NCC-filled PLA shows higher fluorescence intensity in comparison with PLA. The living cells and the dead ones were stained in green and red, respectively. The inclusion of NCC into the scaffold is conducive to cell attachment and proliferation as it denoting low cytotoxicity and good cytocompatibility. The same concept of cell culturing also has been used by Zhang et al. [78]. The difference in this study from the previous study is that the cell culture of hMSCs on the same materials design, PLA and NCC-filled PLA (PLA/NCC) for 14 days. Comparing those two materials designs of the scaffold, more live cells (green stained) on PLA/NCC nanofibrous scaffold than pure PLA. However, few dead cells (red-stained) were also observed. Cell viability and proliferation results suggested that the biocompatibility of PLA was retained by 5% with the additions of NCC. Furthermore, in a study conducted by Zhou et al. [77], the human adipose stem cells (hASCs) were culture on a scaffold composed of PLA/NCC and MPLA/NCC where the NCC content was constant at 5 wt % for 7 days. The results of the cell cultures are shown in Figure 6a–d. It was reported that the MPLA/NCC-5 showed numerous live cells (stained green) than PLA/NCC. Very few dead cells were detected on MPLA/NCC-5 nanofibrous scaffolds. This finding indicated that the cytotoxicity of composites had been reduced during hASC cultivation. This research also conducted an Alamar Blue proliferation viability assay. The assay resulted that the incorporation of NCCs into the scaffold did not contribute to any cytotoxicity effect on hASCs within 7 days, as shown in Figure 6e. The authors have justified that this situation was due to a low amount of NCC included. In future research, there is a need to study the bigger range of NCCs concentration. Yet, the scaffold developed was still able to support cell proliferation and offer good cytocompatibility. The same test is also conducted by many researchers, but by using different cells such as MC3T3 cells [72,85], mouse fibroblast cells [86], and human osteoblast cells [74].

It can be concluded that, from those studies, the inclusion of NCCs as a nanoparticle in bone scaffold enhanced the biocompatibility properties of the scaffold based on the amount of NCCs included. This is because NCCs are naturally derived nanomaterials that originally biocompatible to natural bone. Nevertheless, the incorporation of NCCs has an impact on the cell mechanism during the cell culture and cell viability in vitro. The challenge that arises here is determining the feasible amount of NCCs into the biomaterials that will contribute in term of bone regeneration.

### 4.3. Biodegradability

Biodegradability of scaffold is the ability of the scaffold biomaterials designed to be broken down into simpler substances. Degradation can occur by few mechanisms; enzymatic degradation, hydrolytic degradation and biodegradation. Enzymatic degradation occurs when biomaterials are broken down through the action of enzymes from microorganisms. Meanwhile, hydrolytic degradation involving hydrolysis of biomaterials by water located in host tissue and organ (in vivo). Besides that, biodegradation is caused by cell activity, where the breakdown of biomaterials is due to specific biological activity. Apart from that, the degradation rate is a parameter used to measure the biodegradability of that particular biomaterials. In tissue engineering, the degradation properties of the scaffold are very crucial as it works as a temporary template that assists tissue regeneration and should degrade over time. This is to avoid prolonged allogeneic and xenogeneic reactivity in hostage that would lead to other bad risks. The degradation rate should match the rate of tissue regeneration. If not, the healing process may be incomplete.

The degradation rate of scaffold biomaterials is determined by conducting in vitro or/and in vivo degradation test. The degradation rate is measured by calculating the amount of weight loss over time [87]. Generally, the scaffold is weighed and placed into a sealed container of simulated body fluid (SBF), which is then left to degrade in an incubator at 37 °C (optimum body temperature). Every two weeks, the sample is taken out, washed with distilled water, dried and weighed to calculate the mass loss.

Nanocrystalline cellulose (NCC) is a nanomaterial that has been proven by many studies to offer biodegradability properties and has been widely implemented in scaffold biomaterials. However, the high crystallinity nature and absence of enzyme which could break the glycosidic linkage of NCC in the human body has led to slow or the non-degrading ability of NCC both in vivo and in vitro [88,89]. This condition has been studied by Martson et al. [90], where cellulose-based scaffold has undergone long-term degradation study that resulted in slow degradation in rat subcutaneous after 60 days. In addition, according to Lam et al. [89], for in vivo degradation cases, it has been known that cellulose is degradable. However, in the host body, the resorption of cellulose is unknown to occur, as humans do not synthesize cellulases. This condition is a limitation for the success of cellulose scaffold to be used for in vivo tissue engineering. Not many studies have been focusing on this criterion.

In term of the contribution of NCC inclusion in polymer-based scaffolds, Luo et al. [46] has developed NCC-filled PLA in situ nanocomposite scaffold. From in vitro degradation conducted, the weight loss of scaffold increases with van increase in NCC content. This is due to the presence of NCC has increased the hydrophilicity of scaffolds, so water molecule is more prone to diffusing to ester or other hydrophilic groups, resulting in enhanced hydrolysis of ester groups and breaking the PLA molecular chain. In addition, the inclusion of NCC has improved stability during in vitro degradation. In vitro degradation of scaffolds in PBS medium was evaluated by mass loss in 30 days. The addition of NCC reduced the in vitro degradation rate of MPLA/NCC scaffold in PBS by increasing the crystallinity of MPLA and inhibiting the diffusion of water in polymer matrix even though the addition of NCC resulted in a higher total surface area of scaffolds. Prolonging degradation ability in nanocomposite scaffold by incorporation of NCCs [77]. In contrast, the inclusion of NCC into PVA/n-HA scaffold has reduced the degradation rate as the NCC content increased. This is due to the reduction of salvation and depolymerization [27]. However, theoretically, the hydrophilicity of the biocomposite scaffold increased as the NCC content increased. From here, the selection of biomaterials such as polymers and modification gave different effect on degradation rate of the scaffold.

## 5. Challenge in Future Development of NCC

The extraction process of NCCs could be one of the challenges for future research and development due to the effect of variable factors of the process, which may alter the crystallinity of NCCs, its morphology and the risk of introducing new defects within the NCCs particles. To minimize the defects of the NCCs, there is a need to control the extraction process. The less defect NCCs will retain the higher mechanical integrity and thermal properties of the NCCs. In addition, tighter control of the extraction process on NCC particle size distribution will provide more control on NCC suspensions, NCC-surface modification and NCC-polymer matrix in designing of scaffold materials. In suggestion, optimization and standardization of the extraction process of NCCs should be done in order to control the quality of NCCs produced in terms of process control and variables.

Second, one of the doubting issues about NCC is that its compatibilization properties. As stated in the review, polymer-based biomaterials are broadly used in scaffold materials design. Many works have been conducted to compatibilize the NCCs with polymer matrix, and this issue is still unsolved and needs further study. By increasing the dispersion of NCCs into the polymer, matrices are said will improve the interfacial characteristics of composite and improving the strength and stiffness without sacrificing toughness. However, this step is still a challenge because it has also been reported that an excessive amount of NCCs will degrade the properties of composite materials. Hence, again at this part, an extensive study on optimizing the amount of NCC dispersion should be conducted to produce a good functionalize scaffold.In another aspect, in order to increase the production of NCCs on an industrial scale, the processing cost should be reduced by focusing on ways to increase the yielding of NCCs, but the energy input, chemical usage would be reduced as well as by recycling the used chemical whichever could. The cost reduction will increase the availability of a huge amount of NCCs, which can be supplied into many other applications. To note here, the sources of NCCs should be from agricultural waste as an initiative to turn the waste into something useful.

In terms of tissue engineering application and implementation, there are few challenges that need to be solved. First, from the beginning of the study on tissue engineering, biomaterials for developing the scaffold is the main challenge faced by researchers. This is because the biomaterials must possess all basic requirements properties, which are sufficient mechanical strength, biocompatible and biodegradable, to ensure a successful implementation of scaffolds in the future. Scaffold biomaterials are still in the “trial and error” phase, in which a range of various materials from synthetic to natural sources were studied. There are no materials that fully capture the intricacies of the native tissue nor restore function to an ideal level. One of the efforts is to include the NCCs into synthetic or/and natural biomaterials of scaffolds. However, there is still no clear directions for this effort.

To implement the scaffold in human body, there is still a lack of cell sources. In recent years, the field of stem biology has developed considerably and presents a huge potential for using human originated adult or embryonic stem cells as sources for in vitro generation of tissues. Endothelial cells derived from human embryonic stem cells were shown to generate functional vasculature. However, much more research is required in scaling up these cells and using them for tissue engineering applications. The lack of clinical success may be attributed to several issues. First, the quality or quantity of used cells and preculture conditions of cells seeded onto scaffold are variable or limited. Second, the seeded cells may be subjected to inflammation and nutrient scarcity because of tissue damage and the diffusion of nutrients and oxygen from adjacent vessels.

Therefore, in future developments, all of these challenges must be put into consideration.

## 6. Conclusions

This review is aimed to give a review on the unique properties offered by an organic nanomaterial, macro- or nanocrystalline cellulose for bone tissue engineering applications. Details explanation of macro- or nanocrystalline cellulose with derivation process is included to introduce a basic understanding of this excellent material. The important requirement needed in developing bone tissue engineering scaffolds were elaborated in terms of effects after the inclusion of macro- or nanocrystalline cellulose. There is no doubt that crystalline cellulose is effective to be applied as reinforcing agents or nanofillers in designing the scaffolds materials due to their good performance physiochemically and biocompatibility. The capability of macro- or nanocrystalline cellulose to be modified and easy to be processed has made it an ideal nanomaterial for bone tissue engineering applications, even for other biomedical applications. However, there are still challenges that need to be faced and solved before the scaffold to be applied in humans as real clinical treatments.

## Figures and Tables

**Table 1:** Type of cellulosic particles with their morphology and crystallinity index range.

Type of Cellulose Particles		Sources	Derivation Methods	Particles Size		Crystallinity Index (%)	Ref.
				ℓ (µm)	W (nm)		
Bacterial cellulose (BC)	Bacterial nanocellulose (BNC)	Low molecular weight sugars and alcohols	Bacterial synthesis with the presence of *Gluconacetobacter xylinus*	>1	30–50	65–79	[13,32,48]
Fibrillated cellulose (FC)	Microfibrillated cellulose (MFC)	Wood pulp, potato peel, sugar beet, hemp	Mechanical disintegration produced by high pressure and/or shearing forces of mechanical fibrillated after pretreatment	0.5–50	10–100	51–69	[49]
	Nanofibrillated cellulose (NFC)			0.5–2	4–20	-	[50]
Crystalline cellulose (CC)	Microcrystalline cellulose (MCC)	Cotton, softwood pulp, rice husk, rice straw, wheat straw, empty fruit brunch, ramie, corn stalk, some form of algae and bacteria	Purified cellulosic fibers undergo chemical (acid) hydrolysis after complete dissolution of the non-crystalline fraction	10–50	10–20	80–85	[51]
	Nanocrystalline cellulose (NCC)			0.05–0.5	3–5	54–90	[45,52]

ℓ = length; w = width; ref. = references.

**Table 2:** Recent studies on the contribution of NCCs inclusion in bone scaffold material design in terms of mechanical properties.

Cellulose Sources	Polymer	Additives/Modification	Material Composition	Fabrication Method	Mechanical Properties (MPa)				Ref.
					σ_T_	*E*	σ_C_	*E*	
Cotton	PLA	PLA grafted maleic anhydride (MPLA)	MPLA	Electrospinning	1.6 ± 0.4	7.8 ± 3.1	-	-	[77]
			MPLA/NCC-1		4.8 ± 0.8	77.2 ± 7.8			
			MPLA/NCC-2		4.9 ± 1.0	87.4 ± 8.0			
			MPLA/NCC-5		10.8 ± 1.7	135.1 ± 10.4			
			PLA/NCC-5		6.3 ± 1.2	125.6 ± 9.9			
Cotton	PLA	Add SnCl_2_·H_2_O; p-TSA	PLA	Freeze-drying/lyophilization	-	-	-	19	[46]
			PLA/NCC-0.2					25	
			PLA/NCC-0.4					38	
			PLA/NCC-0.6					65	
			PLA/NCC-0.8					89	
Pine wood	PLA	NCC grafted with peg (CNC-g-PEG)	PLA	Electrospinning	2.8 ± 0.4	-	-	-	[78]
			PLA/NCC-1		2.8 ± 0.5				
			PLA/NCC-5		2.3 ± 0.5				
			PLA/NCC-g-PEG-1		3.5 ± 0.2				
			PLA/NCC-g-PEG-5		4.7 ± 0.3				
			PLA/NCC-g-PEG-10		2.8 ± 0.3				
Sugarcane bagasse	PVA	Incorporated with n-HA	PVA	Freeze-drying	-	-	0.4	0.32	[27]
			PVA/n-HA				0.85	4.68	
			PVA/n-HA/NCC-2				1.39	10.67	
			PVA/n-HA/NCC-4				1.4	10.1	
			PVA/n-HA/NCC-6				1.48	13.41	
			PVA/n-HA/NCC-8				1.6	14.5	
			PVA/n-HA/NCC-10				2.09	16.01	
Commercialized purified cellulose	PVA	Incorporated with ovalbumin (OVA) and n-HA and cross-linked with glutaraldehyde Composition: PVA/OVA/NCC/n-HA	PVA/OVA/NCC/n-HA	Freeze-drying	-	-	-	-	[83]
			1/0.2/0.25/0				0.29	0.37	
			1/0.2/0/2/0.25				0.2	0.46	
			1/0.2/0.15/0.5				0.19	0.92	
			1/0.2/0.1/0.75				0.37	1.2	
			1/0.20/0.05/1				0.25	0.37	
			1/0.2/0/1.25				0.33	0.4	
Commercialized MCC	PVA	-	PVA	Fused deposition modelling (FDM)	11.19	2.88	-	-	[47]
			PVA/NCC-2		11.69	4.25			
			PVA/NCC-5		19.32	4.98			
			PVA/NCC-10		15.46	5.71			
Commercialized MCC	PCL	-	PCL: MCC (1:0)	Fused deposition modelling (FDM)	-	-	-	25	[73]
			PCL: MCC (49:1)					32	
			PCL: MCC (19:1)					29	
			PCL: MCC (9:1)					7	
Softwood sulfite pulp	PCL	Surface oxidation NCC	PCL	Micro-extrusion	10.4 ± 0.9	194.3 ± 12.1	-	-	[72]
			PCL/NCC-1		13.4 ± 1.5	275.2 ± 14.4			
			PCL/NCC-2		15.3 ± 1.0	299.9 ± 15.2			
			PCL/NCC-3		16.3 ± 1.4	353.1 ± 20.9			
			PCL/NCC-5		16.6 ± 0.3	373.8 ± 18.6			
			PCL/NCC-10		18.2 ± 0.3	492.5 ± 44.1			
Wood pulp	-	NCC coated with HAP	NCC	Freeze-drying	-	-	-	80.6 ± 1.4 KPa	[84]
			NCC/HAP at pH 7.4					119.6 ± 2.7 KPa	
			NCC/HAP at pH 8.5					227.6 ± 2.7 KPa	
			NCC/HAP					92.5 ± 2.3 KPa	
Cotton	Chitosan/alginate/HAP	Dicationic crosslinking using CaCl_2_	SC	Freeze-drying	-	-	0.35	-	[53]
			SC/HA				0.38		
			SC/HA/NCC-0.5				0.48		
			SC/HA/NCC-1.0				0.54		
			SC/HA/NCC-2.0				0.51		
Not stated	Silk fibroin (SF)	Incorporated with n-HA	SF	Freeze-drying	-	-	92.1 ± 7.3 KPa	175.2 ± 10.65 KPa	[85]
			SF/NCC				100.8 ± 13.5 KPa	200 ± 12.3 KPa	
			SF/n-HA				140.1 ± 11.4 KPa	428.3 ± 14.4 KPa	
			SF/n-HA/NCC				200.7 ± 15.3 KPa	617.5 ± 25.2 KPa	
Cotton	PLLA	Incorporated with HA pretreatment of particles using a coupling agent	PLLA/HA/MCC	Freeze-drying	-	-	0.5–2.3	8–47	[81]

σ_T_ = tensile strength; σ_C_ = compressive strength; E = elastic modulus.

## Abbreviations

°C	Degree celsius
BC	Bacterial cellulose
BNC	Bacteria nanocellulose
CC	Crystalline cellulose
CNC	Cellulose nanocrystal
DIW	Deionized water
DW	Distilled water
E	Young’s modulus
ECM	Extracellular matrix
FC	Fibrillated cellulose
GPA	Giga pascals
H_2_SO_4_	Sulfuric acid
HA	Hydroxyapatite
hASCs	Human adipose stem cells
HBr	Hydrobromic acid
HCl	Hydrochloric acid
hMSCs	Human mesenchymal stem cells
HNO_3_	Nitric acid
KOH	Potassium hydroxide
LB	Lignocellulosic biomass
M058K	Glioblastoma multiforme cell line
MC3T3	Osteoblast precursor cell line
MCC	Microcrystalline cellulose
MFC	Microfibrillated cellulose
MG63	Human osteosarcoma cell line
MPa	Mega pascals
NaOH	Sodium hydroxide
NCC	Nanocrystalline cellulose
NFC	Nanofibrillated cellulose
n-HA	Nano-hydroxyapatite
NWC	Nanowhisker cellulose
OVA	Ovalbumin
PBS	Phosphate buffered saline
PCL	Ε-poly(caprolactone)
PEG	Poly(ethylene glycol)
PLA	Poly(lactic acid)
PLLA	Poly(l-lactide acid)
PPF	Poly(propylene fumarate)
PVA	Poly(vinyl alcohol)
SBF	Simulated body fluid
TE	Tissue engineering
